# Rehabilitation therapy for a severe case of coronavirus disease 2019: a case report

**DOI:** 10.1186/s13256-022-03559-5

**Published:** 2022-09-02

**Authors:** Toru Takekawa, Kazumi Kashiwabara, Naoki Yamada, Shu Watanabe, Midori Hama, Gentaro Hashimoto, Masahiro Abo, Kyota Shinfuku

**Affiliations:** 1 Department of Rehabilitation Medicine, The Jikei University School of Medicine, 3-25-8, Nishi-Shimbashi, Minato-ku, Tokyo, 105-8461 Japan; 2 Department of Respiratory Medicine, The Jikei University Daisan Hospital, 4-11-1, Izumihoncho, Komae-shi, Tokyo, 201-8601 Japan

**Keywords:** COVID-19, Post-acute COVID-19 syndrome, Intensive care units, Nutrition therapy, Androgenic alopecia, Muscle weakness, Exercise therapy, Rehabilitation, Infection control, Muscle proteins

## Abstract

**Background:**

Patients with severe coronavirus disease 2019 (COVID-19) infection require a long period of time to return to work and society due to significant physical weakness even after recovery. Here we report a patient with a history of nephrectomy who developed severe COVID-19 infection associated with muscle weakness but was able to return to society after rehabilitation therapy.

**Case presentation:**

A Japanese man in his 40s was admitted to the hospital with PCR-based COVID-19 diagnosis. The respiratory condition worsened rapidly and was treated with extracorporeal membrane-assisted ventilation in the intensive case unit. On admission to the Rehabilitation Department on day T + 30 [T: day patient became febrile (38 °C)], he was unable to stand for a long time and used a walker. Rehabilitation therapy was postponed to prevent COVID-19 spread, but the patient was encouraged to exercise during isolation to improve trunk and lower extremity muscle strength. Physical therapy commenced on day T + 49 to improve gait and trunk and lower limb muscle strength. He was able to walk independently and later returned to work following discharge on day T + 53. A computed tomography scan showed an increase in psoas muscle volume from 276 before to 316 cm^3^ after physical therapy, together with a decrease in whole-body extracellular water:total body weight ratio from 0.394 to 0.389.

**Conclusions:**

We have described the beneficial effects of rehabilitation therapy in a patient with severe COVID-19 infection. In addition to exercise, we believe that nutrition is even more important in increasing skeletal muscle mass. Rehabilitation therapy is recommended to enhance the return of severely ill COVID-19 patients to routine daily activity.

## Background

Since the reporting of the first case of severe acute respiratory syndrome coronavirus 2 (SARS-CoV-2) [coronavirus disease 2019 (COVID-19)] in Wuhan, China, in December 2019, the infection has spread across the globe. At the start of 2021, the number of new infections per day per million population was about 76 worldwide [1]. At the same time, a series of reports described mass infections (clusters) of COVID-19 in hospitals and institutional settings in Japan.

Some patients with severe COVID-19 infection who show full recovery experience a significant decline in physical strength after the infection, and early evidence suggests that such patients seem to benefit from rehabilitation therapy, although this is yet to be confirmed [2]. One report [3] described an increase in quadriceps muscle size after respiratory rehabilitation following COVID-19 infection. A few other reports have also described the benefits of rehabilitation therapy after severe COVID-19 infection in Japan and Western countries [4], but there are no reports documenting changes in lower limb skeletal muscle mass by computed tomography (CT).

We describe here a nephrectomized patient with severe COVID-19 infection who required extracorporeal membrane oxygenation (ECMO) during admission to the intensive care unit (ICU) but showed full recovery and returned to society after rehabilitation therapy. While we have reported the same case [5] previously in Japanese, we add here the results of the CT assessment and discuss the effects of nutrition and exercise on skeletal muscle protein synthesis (MPS).

## Case presentation

The patient was a Japanese male in his 40s who had previously suffered renal cell carcinoma and undergone left nephrectomy. Smoking and family histories were both negative. He lived alone in a house surrounded by hills. He used the train for transportation, including taking the train station stairs to the second floor to buy the tickets. He was a private driver of a company car. On T + 3 days [T: day patient became febrile (38 °C)] in 2020, an employee at workplace was diagnosed with COVID-19, and this was the occasion when our patient came in contact with an infected person.

### Present medical history

**Fig. 1:**
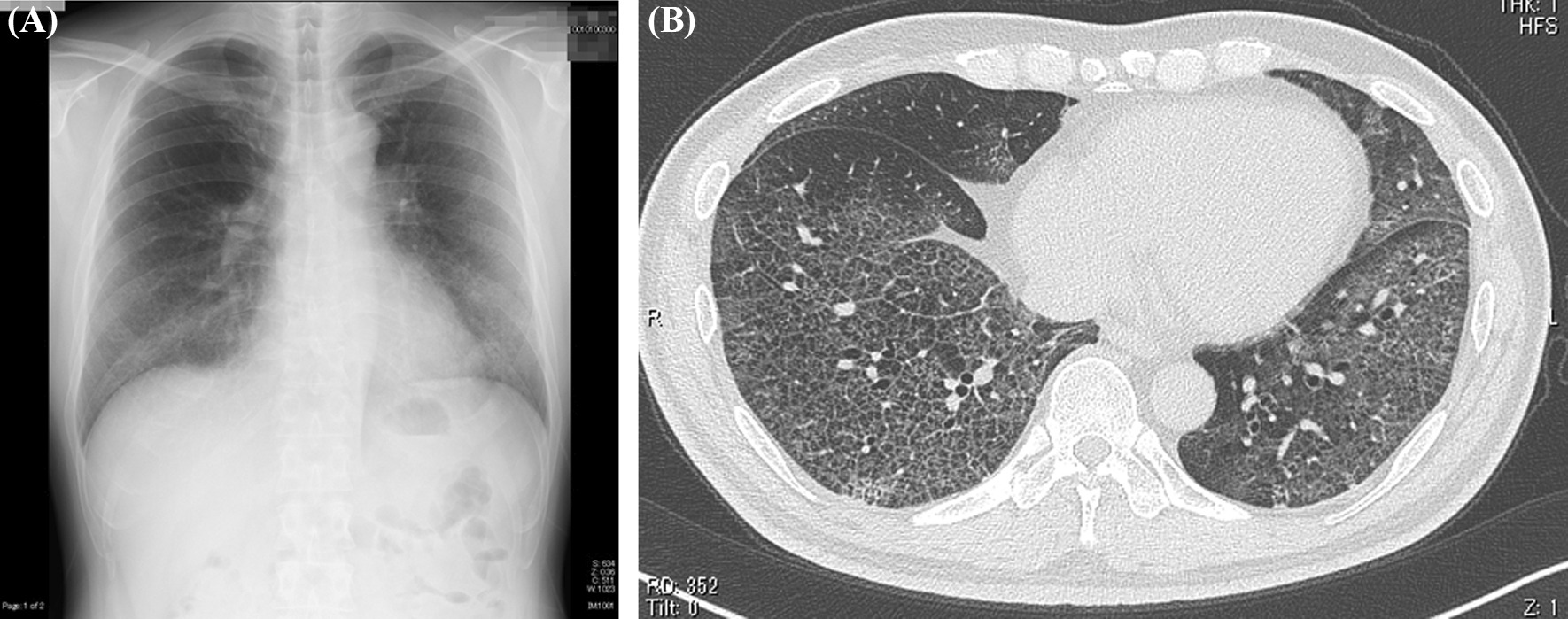
**A** Plain radiograph of the chest (day T + 8). Note the extensive frost shadows in the upper and lower lung fields with bilateral peripheral predominance. **B** Computed tomography image of the chest (day T + 8). Note the broad range of ground-glass opacity with interlobular septal thickening and bilateral peripheral dorsal base predominance.* T* Day patient became febrile (38 °C). Figure is taken from Kashiwabara *et al.* [5], with permission

The patient became febrile (38 °C) on day T in 2020, followed by development of dyspnea on day T + 4; on day T + 6, he decided to visit the emergency room of our hospital where he was checked but sent home the same day after receiving a SARS-CoV-2 PCR test (PCR test). On day T + 8, he returned to the same hospital with worsening dyspnea and general malaise. Blood tests revealed a leukocyte count of 3500/μL; lactose, dehydrogenase (LDH) 646 U/L; C-reactive protein (CRP), 6.32 mg/dL; ferritin, 2291 ng/mL, and a positive PCR test for COVID-19. He was admitted to our hospital. Plain chest X-ray on the same day showed extensive frost shadows in the upper and lower lung fields with bilateral peripheral predominance. Chest CT confirmed these findings, showing a broad range of ground-glass opacity with interlobular septal thickening and bilateral peripheral dorsal base predominance (Fig. 1). Respiratory function deteriorated rapidly after admission, with the patient requiring intubation and mechanical ventilation on day T + 9. He was transferred to another hospital after considering the potential need for ECMO. On day T + 13, he developed aspiration pneumonia while on mechanical ventilation, with further worsening of ventilatory dysfunction, that progressed to bilateral dorsal atelectasis on day T + 14, leading to the initiation of ECMO. He was kept in prone position during the treatment. Following gradual improvement, ECMO was discontinued on day T + 22 as was mechanical ventilation 2 days later. At the last evaluation of the effects of rehabilitation therapy on day T + 29 during the previous hospitalization, the muscle strength was 2 for shoulder abduction, 3 for elbow flexion, 3 for wrist dorsiflexion, 2 for hip flexion, 3 for knee extension, and 3 for ankle dorsiflexion, based on manual muscle testing (MMT). Due to low levels of activities of daily living (ADL), he was transferred back to our hospital on day T + 30 for rehabilitation therapy.

**Fig. 2:**
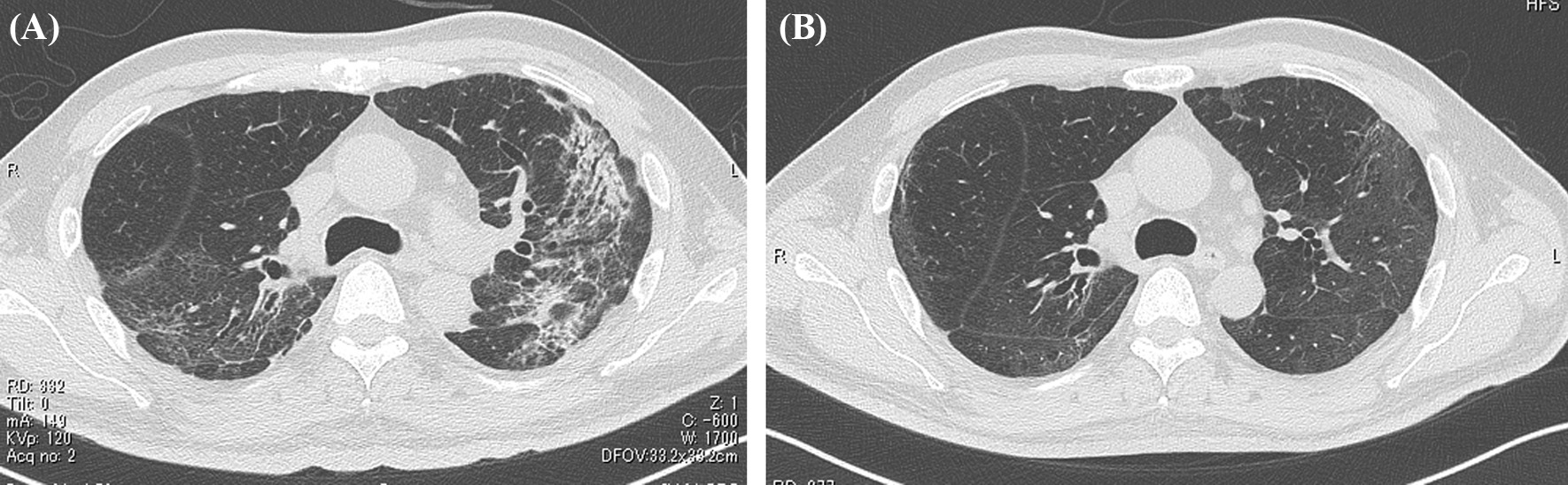
Computed tomography image of the chest. **A** Day T + 30. Note the resolution of the shadows in the bilateral lower lobes, with a band of infiltrating shadows remaining at the periphery of the bilateral upper lobes. **B** Day T + 78. Note the resolution of the band of infiltrating shadows. Figure is taken from Kashiwabara* et al.* [5], with permission

On first admission to our Rehabilitation Department he was given a clinical examination and underwent laboratory tests. These showed a height of 171 cm and body weight of 62 kg; full consciousness; no edema; body temperature, 36.8 °C; heart rate, 86 beats per minute; blood pressure, 114/70 mmHg; respiratory rate, 19 breaths per minute; and oxygen saturation (SpO_2_, 97% (on room air). Arterial blood gas analysis showed pH 7.416, partial pressure CO_2_ (pCO_2_) 30.2 mmHg, and partial pressure O_2_ (pO_2_) 77.7 mmHg. Further tests included those for total protein (6.8 g/dL), albumin (2.9 g/dL), blood urea nitrogen (BUN; 26 mg/dL), creatinine (Cr; 1.32 mg/dL), creatinine clearance (CrCl; 59 mL/minute) (urine analysis at any time), and urine protein (±). Chest CT showed resolution of the shadows in the lower lobes bilaterally, with a band of infiltrating shadows remaining at the periphery of the upper lobes bilaterally (Fig. 2A). MMT showed hip flexion 3/3, knee extension 4/4, knee joint flexion 3/3, foot dorsiflexion 4/4, ankle plantar flexion 4/4, with no muscle weakness in both upper extremities. However, he complained of weakness, such as his cell phone feeling heavy. Cognitive function was normal, and there were no evident clinical abnormalities of the central nervous system, including olfactory or gustatory disturbances, and no neurological abnormalities other than lower limb muscle weakness. While supported, he was able to perform independently basic and orthostatic movements. He experienced a temporary decrease in SpO_2_ to 94% (room air) during the execution of orthostatic movements. The patient could stand for about 1 minute, although he required upper limb support during the task and was unable to hold the position for a long time (about 10 minutes). He used a walker and was able to walk independently for about 10 meters. He was frustrated and worried about COVID-19-related social distancing and restriction of movement introduced during the pandemic. He also complained of compromised motivation during the inactivity.

### Clinical progress and rehabilitation therapy

While the SpO_2_ on room air was within the normal range, the patient complained of marked muscle weakness, mainly in the lower part of the body, together with generalized weakness. Based on the medical history and the generalized and symmetrical muscle weakness reported during the previous hospitalization (provisionally diagnosed at that admission as ICU-acquired weakness (ICU-AW) [6]), we first ruled out conditions associated with muscle weakness. Physical therapy was recommended based on the marked muscle weakness, but could not be provided in line with the hospital policy of withholding such therapy from COVID-19-infected patients until 2 weeks after a negative PCR test. During the 2-week quarantine period, no equipment (diagnostic or rehabilitation therapy) was taken into his hospital room. Instead, he was instructed during this 2-week period to exercise while in isolation, following a self-training program that focused on strengthening the muscles of the trunk, both upper and lower limbs, fingers, and expiratory muscles, including, among other exercises, stretching the trunk, exercising the shoulder girdle and neck, and deep breathing. The patient adhered to the program, which was designed to achieve independence in basic movements and gait, as well as to strengthen respiratory muscles.

The self-exercise program included exercising three times a day, at 1000, 1300, and 1500 hours, for a total of about 30 minutes, three sets at a time, 6 days a week. The patient was not supervised during this exercise-in-isolation program, but was occasionally monitored by nurses or physical therapists. There was no drop in SpO_2_ during the exercising. However, the patient was not monitored with video cameras or remote recording while he remained in isolation. The exercise intensity was increased by the physical therapist after the sixth day. Following this program, the patient was able to walk unassisted by day T + 34 without a walker.

On day T + 37, he was transferred to the general ward and provided with a new self-training program. The aim of this protocol was to improve walking endurance by strengthening trunk and lower limb muscles, such as standing up, standing on one leg, standing on the toes, and squatting. Although he spent most of the time in the hospital room, the patient was gradually introduced to light exercises, such as walking outside the room and inside the hospital. Despite his low endurance, he was able to stand without upper limb support and also on one leg for 10 seconds bilaterally. The measured walking distance while wearing a face mask was 383 m (Table 1). By day T + 39, his body weight had increased to 66.5 kg and spirometry values became normal (Table 1). He was able to do four to five squats by day T + 41, but still had difficulty climbing stairs. When 2 weeks had passed since the negative PCR test, according to the hospital rules at that time the patient was allowed to receive individual therapy (respiratory rehabilitation/physical therapy). In compliance with the standard rules, the patient wore a surgical mask and the therapist wore personal protective equipment. On day T + 49, we started the following individualized rehabilitation therapy, including physical and respiratory rehabilitation therapy, in addition to education on independent training, such as (1) range of motion joint exercises, (2) muscle strengthening exercises, (3) gait exercises, (4) stair climbing exercises, (5) endurance training, and (6) self-directed training, through supervised, non-equipment-based exercises. Endurance was still low, and he suffered from shortness of breath after light jogging for a very short distance. He was later able to go up and down the stairs independently without support, and accordingly was discharged on day T + 53. During the period between hospitalization day T + 30 and day T + 53, the patient was provided with a general diet (1900 kcal/day: 65–75 g protein, 44–61 g fat, 263–311 g carbohydrate, and < 8 g salt), with protein accounting for 15-16% of total calorie intake. At the time of discharge, his body temperature was 36.5 °C; heart rate, 94 beats per minute; blood pressure, 106/70 mmHg; respiratory rate, 14 breaths per minute; SpO_2_, 98% (on room air).

**Table 1:** Results of various motor and respiratory function tests at 1 and 4 months after onset of coronavirus disease 2019 infection

Respiratory function tests	T + 1 month	T + 4 months
VC (L)	3.65	4.43
VC (%)	85.0	104.7
FEV1 (%)	89.7	83.3
DLCO (%)	25.0	50.3
Iliopsoas muscle computed tomography		
Body fat percentage (%)	39.2	37.8
Inner fat rate (%)	39.7	39.8
Psoas major muscle mass (cm^3^)	276.4	316.2
Part of the Psoas major muscle (cm^3^)	98.9	113.4
Bioelectrical impedance analysis^a^		
Body weight (kg)	65.7	71.0
Muscle mass (kg)	46.1	50.1
Body fat mass (kg)	16.9	18.0
ECW/TBW whole (body)^b^	0.394	0.389
ECW/TBW right arm^b^	0.384	0.379
ECW/TBW left arm^b^	0.383	0.378
ECW/TBW right leg^b^	0.400	0.394
ECW/TBW left leg^b^	0.399	0.394
ECW/TBW trunk (of the body)^b^	0.393	0.388
Timed Up and Go test		
Comfortable speed: Clockwise (seconds)	9.0	6.0
Comfortable speed: Left turn (seconds)	10.5	5.9
Maximum speed: Clockwise (seconds)	7.0	6.0
Maximum speed: Left-handed (seconds)	7.4	6.0
Walking for 6 minutes (with mask on)		
Distance walked (m)	383	572
Modified Borg Scale	Shortness of breath 4/fatigue 4	Shortness of breath 3/fatigue 0

Data are from Kashiwabara,* et al.* [5] with some modifications, with permission

*VC* Vital capacity,* FEV1 *forced expiratory volume in the first second,* DLCO* diffusing capacity of the lungs for carbon monoxide, * ECW* extracellular water content,* ICW* intracellular water content,* TBW *total body content (= ICW + ECW),* T* Day patient became febrile (38 °C)

^a^Bioelectrical impedance analysis was performed using an InBody^®^ body composition analyzer (InBody Japan Co., Tokyo, Japan)

^b^ECW/TBW (standard range: 0.360–0.400). In general, a rapid increase in fluid causes an abnormal increase in both ECW and ICW, but the rate of increase in ECW is higher, resulting in a higher ECW/TBW. A decrease in the number of somatic cells that make up the muscle is associated with a decrease in ICW, whereas is ECW/TBW high in the absence of fluid overload

**Fig. 3:**
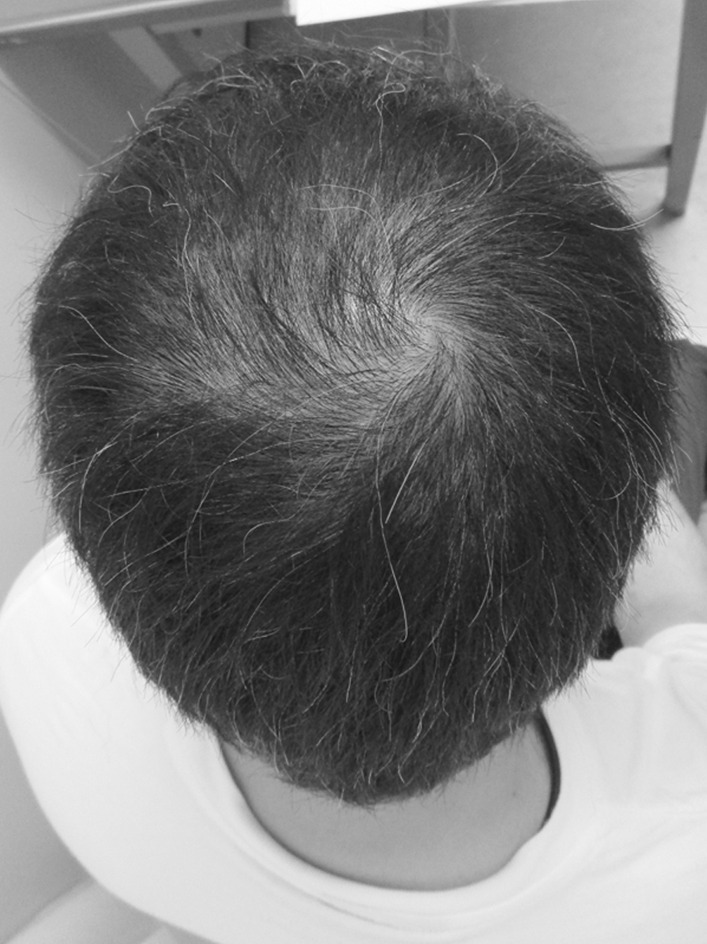
Photograph of the patient illustrating androgenetic alopecia at day T + 100

**Fig. 4:**
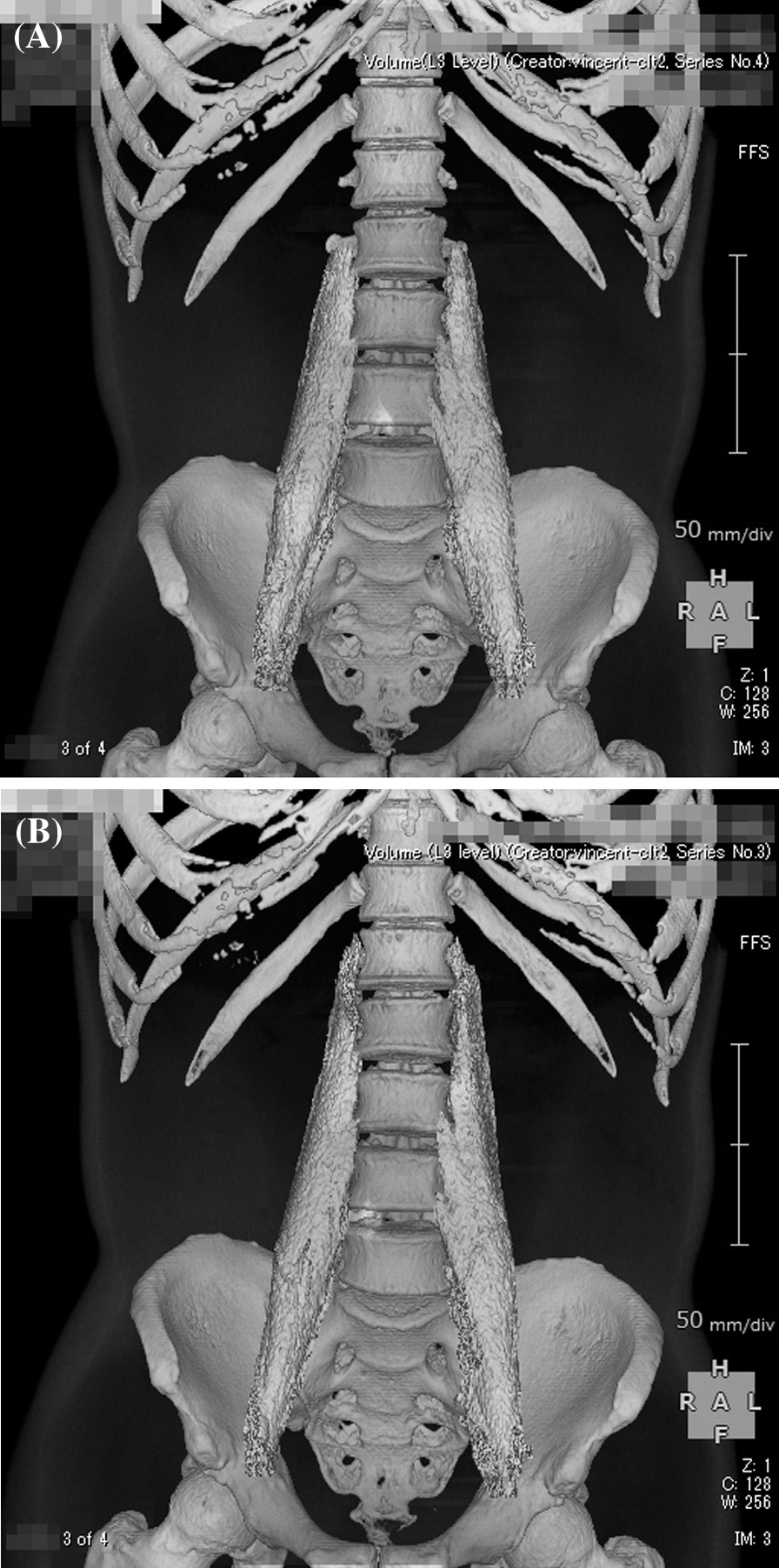
Reconstructed computed tomography images of the psoas major muscle taken at:** A** day T + 41 (muscle mass 276 cm^3^), **B** day T + 116 (muscle mass 316 cm^3^)

On the first post-discharge outpatient visit, he was instructed to continue with the voluntary training program, with a subjective aerobic exercise intensity of “easy,” resistance exercise intensity of 8–12 RM (Repetition Maximum), and with no restrictions on exercise type. He was also instructed to gradually increase the load with the recovery of physical strength. Based on his good general condition and renal function, the patient was encouraged to increase the intensity of exercise and consume a protein-rich diet after discharge, including natto (fermented soybeans) and chicken meat. A chest CT taken on day T + 78 showed resolution of the band of infiltrating shadows (Fig. 2B). Laboratory tests at this same time point showed total protein of 6.7 g/dL, albumin of 4.0 g/dL, BUN of 18 mg/dL, Cr of 1.36 mg/dL, CrCl of 61 mL/minute, and ± proteinuria. He returned to work on day T + 88 and continued to show improvement in physical fitness, with a body weight gain to 72 kg (75 kg before COVID-19). However, hair loss was noted after day T + 100 (Fig. 3), together with a transverse nail groove. Table 1 shows the changes in various parameters recorded at 1 and 4 months after the onset of COVID-19 (day T). Improvements were noted in respiratory function tests, the Timed Up and Go test (TUG), and 6-minute walking test [7], with a decrease in the ECW/TBW ratio from 0.394 (day T + 52) to 0.389 (day T + 120), as measured by bioelectrical impedance analysis using the In Body^®^ (InBody Japan Co., Tokyo, Japan) body composition analyzer, suggesting an increase in the number of somatic cells that make up muscles. In fact, the volume of the psoas muscle, as measured by CT, increased from 276 cm^3^ (day T + 41) to 316 cm^3^ (day T + 116) (Fig. 4A, B). Respiratory and motor functions recovered with no complications.

## Discussion

This patient required treatment with ECMO for severe COVID-19, and later showed full recovery after rehabilitation therapy.

### COVID-19 and rehabilitation therapy

In COVID-19 infections, the mechanism of weakness is multifactorial, including decline in exercise endurance associated with cardiopulmonary dysfunction and muscle atrophy associated with long-term immobilization [8]. Especially for patients with severe COVID-19, return to normal life after discharge from the hospital is often difficult [8]. In addition, since the majority of COVID-19 patients have other comorbidities, these can exacerbate the general condition after discharge [8].

Post-acute COVID-19 syndrome (PACS) [9] is defined as persistent symptoms > 3–4 weeks after the onset of COVID-19. Our patient was diagnosed with PACS based on the presence of fatigue, muscle weakness, and hair loss, even though he did not exhibit other clinical features of the syndrome [for example, dyspnea, cough, depression, cognitive impairment (brain fog), and palpitation] [10]. At discharge, he was completely free of fatigue and muscle weakness. We believe that muscle weakness in the subacute phase of COVID-19 is largely due to ICU-AW [6] in critically ill patients who have been placed on ventilators or ECMO, and is largely due to disuse syndrome, especially in the elderly. When our patient was transferred to our hospital, he had lower limb muscle weakness and was presumed to be recovering from ICU-AW [6]. The term ICU-AW designates clinically detected weakness in critically ill patients in whom there is no plausible etiology other than critical illness [6].

Rehabilitation offers various benefits, both during the acute stage and especially during recovery [8]. Zhao *et al.* [11] recommended starting physiotherapy during the acute phase and to continue it after transfer to the rehabilitation ward. Another randomized controlled trial (RCT) [12] showed significant improvement in respiratory function, endurance, quality of life (QoL), and depression following two sessions of 6-week, 10-minute/week respiratory rehabilitation following discharge from acute care. A recent study showed that physical therapy, in addition to respiratory physical therapy, provides reconditioning and physical activity in post-acute patients with COVID-19 [13]. Respiratory and mild aerobic training for patients who had mild COVID-19 is desirable [8]. One study recommended keeping the metabolic rate of aerobic exercise to < 3 Mets at the beginning of therapy [7]. In our case, rehabilitation therapy was conducted at a similar exercise intensity.

While it is important to implement quarantine and isolation measures to prevent the spread of the disease, there is also a need to implement physical exercise during this period to protect against catabolic crisis [14]. The effects of physical therapy on PACS remain to be fully understood, but patients with PACS should continue to participate in rehabilitation therapy (if medically approved) similar to those with ICU-AW [6] or disuse syndrome as early as possible.

### Infection control during rehabilitation therapy

When implementing rehabilitation therapy for persons infected with COVID-19, it is important to take measures that prevent the spread of infection, including isolation of the patients. In Japan, the “first wave” of infection spread with the B.1.1 strain of SARS-CoV-2 that occurred around day T in 2020 [15]. For our patient, after careful consideration at the time of uncertainty about the duration of the virus' infectivity, it was decided to provide custom-tailored rehabilitation therapy at 2 weeks after testing negative on the PCR test for SARS-CoV-2. To prevent the spread of coronavirus infection in the hospital through therapists in contact with the patient, we provided the patient with supervised and non-instrumental exercise, focusing mainly on the trunk and lower limbs. However, the use of neuromuscular electrical stimulation for muscle strengthening is generally recommended [7]. The patient was kept in isolation in a private room for a relatively long period of time after recovery from COVID-19 infection, resulting in a significant reduction of physical strength. Voluntary training was implemented on day T + 31 and rehabilitation therapy started on day T + 49.

Since February 2021, we now start individualized therapy during the observation period of patient isolation, immediately after the second negative result by the rapid transcription-reverse transcription concerted reaction (TRC) method [16], because it is especially important to take precautions against nosocomial infection. Other common viral infection control measures used in our department include checking patients and staff for fever and cold symptoms, selecting appropriate personal protective equipment for staff depending on the risk of infection during physical and occupational therapy and speech and feeding therapy, cleaning equipment after use, and ventilating the rehabilitation room. In addition to these measures, we have also applied the following precautions: introduction of a ward-assignment system for therapists to facilitate the tracing of infection routes; the separation of flow lines between inpatients and outpatients; and a review of the needs and frequency of rehabilitation therapies for patients (including ensuring physical distance). As of January 2022, rehabilitation therapy is provided to patients who are COVID-19-positive following the recommendation for such therapy by the physician. For our patient, we prepared a self-training instruction sheet, but in the future we are considering on-demand videos and online (simultaneous interactive) instructions. We believe that measures to protect against infection in the provision of rehabilitation therapy should be flexible and modified according to the situation.

### Exercise and nutrition, and their relation in muscle protein synthesis (MPS)

The relatively rapid recovery of muscle strength during the subacute phase in our patient was presumed to be due to an increase in the number of active motor units and synchronization of the mobilization of multiple motor units, rather than to an increase in muscle mass. We also presume that the regain of reduced skeletal muscle mass observed at follow-up was related to the improvement in physical fitness through daily routine tasks, commuting, and work. Our data showed improvement in skeletal muscle mass at 4 months after the onset of the infection, as measured by CT imaging (Fig. 4A, B). In our case, increased skeletal muscle mass, through increased number of muscle fibers, was also inferred from the significant increase in body weight, lack of general edema, increased ICW, and decreased ECW/TBW ratio by the BIA (Table 1). Previous studies indicated that skeletal muscle mass/body mass index correlates with gait speed, ADL, frailty, and functional and disability measures [17]. We believe that the increase in skeletal muscle mass in our patient was related to his relatively young age, motivation to improve his general condition, proper nutrition, and voluntary exercise regimen during hospitalization and after discharge.

Few RCTs have shown that exercise also increases muscle mass in patients with sarcopenia [18]. Exercise programs, especially those based on resistance training, can improve muscle strength and physical performance, but are not associated with increased muscle mass [18]. Our patient showed good improvement of motor function (Table 1). One month after the onset, the TUG on comfort speed was around 10 seconds, which was close to the cutoff value [19] for sarcopenia, but the value improved markedly at 4 months after onset.

Based on this case, we believe that exercise is important for increasing skeletal muscle mass, although nutrition is even more important. In this regard, evidence indicates that mammalian target of rapamycin (mTOR) plays an important role in the control of muscle mass [20]. Previous studies [21] have shown that resistance exercise increases muscle protein turnover due to enhanced synthesis and degradation. Given that exercise sensitizes skeletal muscles to the anabolic effect of protein ingestion, early rehabilitation may act synergistically with dietary protein to protect muscle mass and function during post-injury disuse conditions [22]. Maintenance of skeletal muscle mass is regulated by a balance between anabolic and catabolic processes. mTOR controls the anabolic and catabolic signaling of skeletal muscle mass, resulting in modulation of muscle hypertrophy and muscle wastage [20]. It has also been shown that mTOR signaling, which controls protein synthesis, is reduced in pulmonary inflammation [23]. These findings suggest that ICU-AW [6] encompasses resistance to muscle anabolism and increased catabolism. Among the branched-chain amino acids (BCAAs), leucine is the primary stimulant of mTOR-mediated muscle synthesis [24]. In order to increase skeletal muscle mass after ICU-AW [6], we believe that nutrition as the "source" for muscle protein is even more important than exercise as a trigger to promote this increase, and among the BCAAs, leucine plays an key role. Although our patient did not take dietary supplements of BCAAs, he consumed chicken breast meat, which contains relatively high amounts of leucine. As in other similar conditions, a long ICU stay may *per se* directly worsen or cause malnutrition in COVID-19 patients, negatively affecting skeletal muscle mass and function, and potentially lead to disability, poor QoL, and additional morbidity. Prevention, diagnosis, and treatment of malnutrition should therefore be routinely included in the overall assessment of severely ill COVID-19 patients [25].

## Conclusions

We provided rehabilitation therapy for a middle-aged man during recovery from severe COVID-19 infection, similar to our protocol for ICU-AW [6], focusing on education on nutritional intake, voluntary training, and supervised exercise mainly of the trunk and lower limbs. Such management enhanced QoL and return to normal routine ADL.

We have described the findings of a single case of severe COVID-19, with emphasis on the role of physical rehabilitation, including nutrition, in facilitating the rapid return of patients infected with COVID-19 to their normal life. The collecting and reporting of more similar cases will help establish the true benefits of physical therapy combined with a high-protein diet on the speedy recovery from COVID-19 infection.

We were particularly cautious in providing the patient with individual therapy at the start of the pandemic. However, we are now beginning to see an “exit” from the COVID-19 pandemic. It is possible that the pandemic will become endemic, with the emergence of mutated strains and repeated epidemics. There is no doubt that safer vaccines with fewer adverse reactions will be developed soon, together with better oral medications. In our opinion, it is important to benefit from this experience to provide rehabilitation therapy in a flexible and appropriate manner in response to the ever-changing infectious situation in the society when new unknown infectious diseases occur.

## Data Availability

The datasets generated and/or analyzed during the current study are not publicly available due the perspective of protecting personal information but are available from the corresponding author on reasonable request.

## Abbreviations

ADL: Activities of daily living
BIA: Bioelectrical impedance analysis
CrCl: Creatinine clearance
CT: Computed tomography
COVID-19: Coronavirus disease 2019
ECMO: Extracorporeal membrane oxygenation
ECW: Extracellular water
ICU: Intensive care unit
ICU-AW: ICU-acquired weakness
MMT: Manual muscle testing
MPS: Muscle protein synthesis
mTOR: Mammalian target of rapamycin
PACS: Post-acute COVID-19 syndrome
QoL: Quality of life
RCT: Randomized controlled trial
SARS-CoV-2: Severe acute respiratory syndrome coronavirus 2
TBW: Total body water
TUG: Timed Up and Go test
